# 3-(4-Cyano­phen­yl)-*N*-phenyl­oxirane-2-carboxamide

**DOI:** 10.1107/S1600536810043059

**Published:** 2010-10-30

**Authors:** Long He

**Affiliations:** a College of Chemistry and Chemical Engineering, China West Normal University, Nanchong 637002, People’s Republic of China

## Abstract

The asymmetric unit of the crystal structure of the title compound, C_16_H_12_N_2_O_2_, contains two independent mol­ecules. In each mol­ecule, the two aromatic rings adopt a *cis* configuration about the central epoxide ring, and are oriented at dihedral angles of 61.5 (5) and 74.4 (5)°with respect to the epoxide ring in one mol­ecule, and 60.1 (5) and 72.1 (5)° in the other one. Inter­molecular classical N—H⋯O and weak C—H⋯O hydrogen bonds are present in the crystal structure.

## Related literature

For the use of epoxide-containing compounds as building blocks in synthesis, see: Diez *et al.* (2008 ▶); Porter & Skidmore (2000 ▶); Shing *et al.* (2006 ▶); Zhu & Espenson (1995 ▶). For related structures, see: He (2009 ▶); He & Chen (2009 ▶).
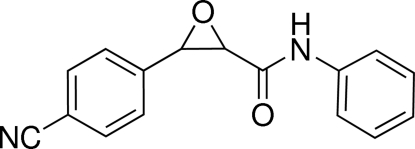


## Experimental

### 

#### Crystal data

C_16_H_12_N_2_O_2_
*M* *_r_* = 264.28
Monoclinic, 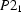

*a* = 5.1332 (1) Å
*b* = 18.0803 (6) Å
*c* = 15.0202 (4) Å
β = 90.449 (2)°
*V* = 1393.98 (7) Å^3^
*Z* = 4
Cu *K*α radiation
μ = 0.69 mm^−1^
*T* = 293 K
0.36 × 0.34 × 0.30 mm

#### Data collection

Oxford Diffraction Gemini S Ultra diffractometer
Absorption correction: multi-scan (*CrysAlis PRO*; Oxford Diffraction, 2009 ▶) *T*_min_ = 0.790, *T*_max_ = 0.820
14057 measured reflections
2843 independent reflections
2534 reflections with *I* > 2σ(*I*)
*R*_int_ = 0.027

#### Refinement

*R*[*F* ^2^ > 2σ(*F* ^2^)] = 0.083
*wR*(*F* ^2^) = 0.178
*S* = 1.02
2843 reflections
322 parameters
13 restraints
H atoms treated by a mixture of independent and constrained refinement
Δρ_max_ = 0.23 e Å^−3^
Δρ_min_ = −0.21 e Å^−3^

### 

Data collection: *CrysAlis CCD* (Oxford Diffraction, 2008 ▶); cell refinement: *CrysAlis RED* (Oxford Diffraction, 2008 ▶); data reduction: *CrysAlis RED*; program(s) used to solve structure: *SHELXS97* (Sheldrick, 2008 ▶); program(s) used to refine structure: *SHELXL97* (Sheldrick, 2008 ▶); molecular graphics: *ORTEP-3* (Farrugia, 1997) ▶; software used to prepare material for publication: *SHELXL97*.

## Supplementary Material

Crystal structure: contains datablocks global, I. DOI: 10.1107/S1600536810043059/xu5057sup1.cif

Structure factors: contains datablocks I. DOI: 10.1107/S1600536810043059/xu5057Isup2.hkl

Additional supplementary materials:  crystallographic information; 3D view; checkCIF report

## Figures and Tables

**Table 1:** Hydrogen-bond geometry (Å, °)

*D*—H⋯*A*	*D*—H	H⋯*A*	*D*⋯*A*	*D*—H⋯*A*
N1—H6⋯O1^i^	0.90 (4)	2.21 (3)	2.960 (6)	141 (4)
N3—H22⋯O4^ii^	0.89 (3)	2.09 (3)	2.923 (5)	157 (4)
C8—H8⋯O3^iii^	0.98	2.49	3.287 (8)	138
C15—H15⋯O1^i^	0.93	2.58	3.505 (6)	171
C24—H24⋯O2^iv^	0.98	2.54	3.370 (7)	142

Symmetry codes: (i) 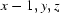; (ii) 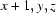; (iii) 
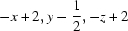
; (iv) 
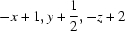
.

**Fig. 1.**
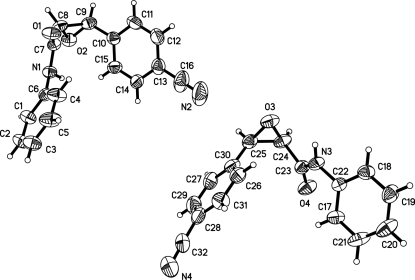
The molecular structure of (I) with 30% probability displacement ellipsoids (arbitrary spheres for H atoms).

## supplementary crystallographic information

### Comment

Epoxides are particularly versatile synthetic intermediates which can readily be converted into a wide range of polyfunctional compounds (Diez *et al.*, 2008; Porter *et al.*, 2000; Shing *et al.*, 2006). A useful method for the synthesis of α, β-epoxy carbonyl compounds and related compounds is the Darzens condensation (Zhu *et al.*, 1995). We report herein the crystal structure of the title compound.

The molecular structure of (I) is shown in Fig. 1. Bond lengths and angles in (I) are normal. The asymmetric unit of the title compound consists of two crystallographically independent molecules (Fig. 1), each of which adopts a *cis* configuration about the epoxides ring. The dihedral angle between the C1—C6 and C10—C15 ring is 44.80 (21)° and that between C17—C22 and C26–31 phenyl ring is 47.93 (18)°. Epoxide ring O2—C8/C9 makes dihedral angles of 61.48 (35)° and 74.38 (27)° with phenyl rings C1—C6 and C10—C15, respectively. Epoxide ring O3—C24/C25 makes dihedral angles of 60.09 (36)° and 72.09 (31)° with phenyl rings C17—C22 and C26—C31, respectively. The crystal packing is stabilized by N—H···0 and C—H···0 hydrogen bonding (Table 1).

### Experimental

2-Chloro-*N*-phenylacetamide (0.17 g, 1.0 mmol) and potassium hydroxide (0.112 g, 2.0 mmol) were dissolved in acetonitrile (2 ml). To the solution was added 4-cyanophenylaldehyde (0.131 g, 1.0 mmol) at 298 K, the solution was stirred for 60 min and removal of solvent under reduced pressure, the residue was purified through column chromatography. Single crystals suitable for X-ray diffraction were obtained by slow evaporation of an ethyl acetate solution at room temperature for 1 d.

### Refinement

H atoms on N atoms were located in a difference Fourier map and refined isotropically. Other H atoms were placed in calculated positions with C—H = 0.93–0.98 Å, and refined using a riding model with *U*_iso_(H) = 1.2*U*_eq_(C). As no significant anomalous scatterings, Friedel pairs were merged.

### Crystal data

**Table:**

C_16_H_12_N_2_O_2_	*F*(000) = 552
*M**_r_* = 264.28	*D*_x_ = 1.259 Mg m^−^^3^
Monoclinic, *P*2_1_	Cu *K*α radiation, λ = 1.54184 Å
Hall symbol: P 2yb	Cell parameters from 8634 reflections
*a* = 5.1332 (1) Å	θ = 2.4–72.1°
*b* = 18.0803 (6) Å	µ = 0.69 mm^−^^1^
*c* = 15.0202 (4) Å	*T* = 293 K
β = 90.449 (2)°	Block, colorless
*V* = 1393.98 (7) Å^3^	0.36 × 0.34 × 0.30 mm
*Z* = 4	

### Data collection

**Table:**

Oxford Diffraction Gemini S Ultra diffractometer	2843 independent reflections
Radiation source: fine-focus sealed tube	2534 reflections with *I* > 2σ(*I*)
graphite	*R*_int_ = 0.027
Detector resolution: 15.9149 pixels mm^-1^	θ_max_ = 73.4°, θ_min_ = 2.9°
ω scans	*h* = −4→6
Absorption correction: multi-scan (*CrysAlis PRO*; Oxford Diffraction, 2009)	*k* = −22→21
*T*_min_ = 0.790, *T*_max_ = 0.820	*l* = −18→18
14057 measured reflections	

### Refinement

**Table:**

Refinement on *F*^2^	Secondary atom site location: difference Fourier map
Least-squares matrix: full	Hydrogen site location: inferred from neighbouring sites
*R*[*F*^2^ > 2σ(*F*^2^)] = 0.083	H atoms treated by a mixture of independent and constrained refinement
*wR*(*F*^2^) = 0.178	*w* = 1/[σ^2^(*F*_o_^2^) + (0.019*P*)^2^ + 2.850*P*] where *P* = (*F*_o_^2^ + 2*F*_c_^2^)/3
*S* = 1.02	(Δ/σ)_max_ < 0.001
2843 reflections	Δρ_max_ = 0.23 e Å^−^^3^
322 parameters	Δρ_min_ = −0.21 e Å^−^^3^
13 restraints	Extinction correction: *SHELXL97* (Sheldrick, 2008), Fc^*^=kFc[1+0.001xFc^2^λ^3^/sin(2θ)]^-1/4^
Primary atom site location: structure-invariant direct methods	Extinction coefficient: 0.0029 (3)

### Special details

**Table:**

Geometry. All e.s.d.'s (except the e.s.d. in the dihedral angle between two l.s. planes) are estimated using the full covariance matrix. The cell e.s.d.'s are taken into account individually in the estimation of e.s.d.'s in distances, angles and torsion angles; correlations between e.s.d.'s in cell parameters are only used when they are defined by crystal symmetry. An approximate (isotropic) treatment of cell e.s.d.'s is used for estimating e.s.d.'s involving l.s. planes.
Refinement. Refinement of *F*^2^ against ALL reflections. The weighted *R*-factor *wR* and goodness of fit *S* are based on *F*^2^, conventional *R*-factors *R* are based on *F*, with *F* set to zero for negative *F*^2^. The threshold expression of *F*^2^ > σ(*F*^2^) is used only for calculating *R*-factors(gt) *etc*. and is not relevant to the choice of reflections for refinement. *R*-factors based on *F*^2^ are statistically about twice as large as those based on *F*, and *R*- factors based on ALL data will be even larger.

### Fractional atomic coordinates and isotropic or equivalent isotropic displacement parameters (Å^2^)

**Table:**

	*x*	*y*	*z*	*U*_iso_*/*U*_eq_	
O2	0.6391 (8)	0.2940 (3)	1.1787 (3)	0.0789 (13)	
O3	0.9368 (8)	0.7460 (3)	0.6402 (3)	0.0897 (15)	
O4	0.4072 (7)	0.8269 (3)	0.5190 (3)	0.0903 (16)	
N3	0.8436 (8)	0.8319 (3)	0.4904 (3)	0.0638 (13)	
C10	0.7910 (7)	0.41180 (18)	1.1080 (2)	0.0678 (16)	
C11	0.9688 (7)	0.4697 (2)	1.1039 (3)	0.086 (2)	
H11	1.0993	0.4738	1.1469	0.103*	
C12	0.9516 (9)	0.5213 (2)	1.0355 (3)	0.095 (2)	
H12	1.0705	0.5600	1.0328	0.114*	
C13	0.7566 (11)	0.5150 (2)	0.9712 (3)	0.084 (2)	
C14	0.5788 (9)	0.4571 (3)	0.9753 (3)	0.090 (2)	
H14	0.4483	0.4529	0.9323	0.108*	
C15	0.5960 (7)	0.4055 (2)	1.0437 (3)	0.080 (2)	
H15	0.4770	0.3668	1.0465	0.095*	
O1	1.1682 (7)	0.2549 (3)	1.0279 (3)	0.0840 (14)	
N1	0.7379 (8)	0.2356 (3)	1.0097 (3)	0.0624 (12)	
C1	0.5444 (6)	0.1679 (2)	0.8919 (2)	0.0757 (19)	
H1	0.4307	0.1479	0.9333	0.091*	
C2	0.5285 (8)	0.1465 (2)	0.8031 (2)	0.092 (2)	
H2	0.4042	0.1121	0.7851	0.111*	
C3	0.6986 (9)	0.1765 (3)	0.7412 (2)	0.090 (2)	
H3	0.6879	0.1621	0.6819	0.108*	
C5	0.8845 (8)	0.2279 (3)	0.7681 (2)	0.106 (3)	
H5	0.9982	0.2479	0.7267	0.128*	
C4	0.9004 (8)	0.2493 (3)	0.8568 (3)	0.090 (2)	
H4	1.0248	0.2837	0.8748	0.108*	
C6	0.7304 (7)	0.2194 (2)	0.9187 (2)	0.0599 (15)	
C23	0.6321 (11)	0.8126 (4)	0.5387 (4)	0.0669 (16)	
C7	0.9480 (10)	0.2563 (4)	1.0554 (4)	0.0646 (15)	
C9	0.8289 (13)	0.3528 (3)	1.1764 (4)	0.078 (2)	
H9	0.8946	0.3695	1.2344	0.093*	
C8	0.8993 (12)	0.2764 (3)	1.1510 (4)	0.0728 (18)	
H8	1.0050	0.2495	1.1949	0.087*	
C25	0.6995 (13)	0.7002 (3)	0.6400 (4)	0.0759 (19)	
H25	0.6463	0.6826	0.6989	0.091*	
C24	0.6896 (12)	0.7803 (4)	0.6287 (4)	0.077 (2)	
H24	0.6255	0.8085	0.6798	0.092*	
C32	0.5095 (19)	0.4971 (4)	0.3637 (5)	0.112 (3)	
C31	0.7737 (7)	0.5940 (2)	0.4233 (2)	0.086 (2)	
H31	0.8760	0.5939	0.3725	0.104*	
C26	0.8234 (7)	0.6446 (2)	0.4910 (3)	0.0772 (19)	
H26	0.9591	0.6784	0.4855	0.093*	
C30	0.6705 (9)	0.6448 (3)	0.5670 (2)	0.0753 (19)	
C27	0.4677 (9)	0.5943 (3)	0.5752 (3)	0.086 (2)	
H27	0.3654	0.5944	0.6260	0.104*	
C29	0.4180 (8)	0.5438 (3)	0.5075 (3)	0.099 (2)	
H29	0.2823	0.5100	0.5130	0.118*	
C28	0.5709 (9)	0.5436 (2)	0.4316 (3)	0.085 (2)	
C17	0.6504 (6)	0.8529 (2)	0.3446 (2)	0.0733 (19)	
H17	0.5233	0.8178	0.3569	0.088*	
C22	0.8404 (7)	0.8694 (2)	0.40799 (19)	0.0563 (14)	
C18	1.0305 (7)	0.9219 (2)	0.3896 (3)	0.0715 (18)	
H18	1.1576	0.9329	0.4321	0.086*	
C19	1.0306 (8)	0.9579 (2)	0.3079 (3)	0.089 (2)	
H19	1.1578	0.9930	0.2956	0.107*	
C20	0.8407 (9)	0.9414 (3)	0.2444 (2)	0.102 (3)	
H20	0.8407	0.9655	0.1897	0.122*	
C21	0.6506 (7)	0.8889 (3)	0.2628 (2)	0.100 (3)	
H21	0.5235	0.8779	0.2204	0.120*	
C16	0.736 (2)	0.5652 (4)	0.9039 (5)	0.131 (4)	
N4	0.451 (2)	0.4589 (5)	0.3060 (6)	0.147 (3)	
N2	0.723 (3)	0.6062 (5)	0.8458 (6)	0.184 (5)	
H6	0.598 (6)	0.261 (3)	1.028 (3)	0.071 (18)*	
H22	1.001 (5)	0.833 (3)	0.515 (3)	0.058 (15)*	

### Atomic displacement parameters (Å^2^)

**Table:**

	*U*^11^	*U*^22^	*U*^33^	*U*^12^	*U*^13^	*U*^23^
O2	0.079 (3)	0.099 (3)	0.058 (2)	−0.004 (3)	0.0072 (19)	0.001 (2)
O3	0.084 (3)	0.123 (4)	0.062 (2)	−0.001 (3)	−0.016 (2)	0.011 (3)
O4	0.051 (2)	0.139 (4)	0.081 (3)	0.003 (3)	−0.0005 (19)	0.030 (3)
N3	0.047 (2)	0.091 (3)	0.054 (2)	0.005 (2)	−0.0022 (19)	0.010 (2)
C10	0.073 (3)	0.069 (4)	0.062 (3)	0.007 (3)	−0.001 (3)	−0.014 (3)
C11	0.080 (4)	0.085 (5)	0.094 (5)	−0.013 (4)	−0.012 (4)	−0.016 (4)
C12	0.115 (6)	0.071 (4)	0.100 (5)	−0.013 (4)	−0.003 (5)	−0.003 (4)
C13	0.106 (5)	0.070 (4)	0.078 (4)	0.008 (4)	0.008 (4)	−0.004 (3)
C14	0.128 (6)	0.066 (4)	0.075 (4)	0.016 (4)	−0.025 (4)	−0.009 (3)
C15	0.087 (4)	0.081 (4)	0.071 (4)	0.009 (4)	−0.016 (3)	−0.009 (4)
O1	0.0504 (19)	0.129 (4)	0.072 (2)	−0.011 (2)	−0.0029 (18)	−0.015 (3)
N1	0.050 (2)	0.079 (3)	0.059 (2)	0.001 (2)	0.0008 (19)	−0.005 (2)
C1	0.065 (3)	0.089 (4)	0.074 (4)	−0.011 (3)	0.004 (3)	−0.021 (4)
C2	0.095 (5)	0.100 (5)	0.083 (4)	−0.012 (4)	−0.008 (4)	−0.032 (4)
C3	0.089 (4)	0.115 (6)	0.065 (4)	−0.006 (4)	0.002 (3)	−0.020 (4)
C5	0.099 (5)	0.165 (8)	0.055 (3)	−0.029 (5)	−0.001 (3)	0.013 (5)
C4	0.079 (4)	0.128 (6)	0.062 (3)	−0.032 (4)	−0.002 (3)	0.005 (4)
C6	0.048 (2)	0.070 (4)	0.062 (3)	0.005 (3)	0.002 (2)	0.004 (3)
C23	0.058 (3)	0.078 (4)	0.066 (3)	−0.006 (3)	−0.004 (3)	0.008 (3)
C7	0.048 (3)	0.069 (3)	0.076 (3)	0.005 (3)	−0.011 (2)	0.000 (3)
C9	0.084 (4)	0.100 (5)	0.050 (3)	0.003 (4)	−0.003 (3)	−0.001 (3)
C8	0.070 (3)	0.096 (5)	0.052 (3)	0.009 (3)	−0.010 (3)	0.003 (3)
C25	0.080 (4)	0.091 (5)	0.057 (3)	−0.012 (4)	−0.001 (3)	0.019 (3)
C24	0.070 (3)	0.113 (5)	0.048 (3)	0.013 (4)	0.002 (3)	0.011 (3)
C32	0.159 (8)	0.061 (4)	0.116 (5)	−0.003 (5)	−0.014 (6)	0.004 (4)
C31	0.087 (4)	0.091 (5)	0.082 (5)	0.003 (4)	0.011 (4)	−0.001 (4)
C26	0.077 (4)	0.087 (5)	0.067 (4)	0.004 (4)	0.006 (3)	0.011 (4)
C30	0.077 (4)	0.090 (5)	0.058 (3)	0.002 (4)	−0.011 (3)	0.018 (3)
C27	0.098 (5)	0.080 (4)	0.082 (4)	−0.004 (4)	0.014 (4)	0.026 (4)
C29	0.103 (5)	0.076 (5)	0.116 (5)	−0.012 (4)	0.004 (4)	0.020 (4)
C28	0.109 (5)	0.067 (4)	0.080 (4)	0.016 (4)	−0.009 (3)	0.018 (3)
C17	0.065 (3)	0.100 (5)	0.055 (3)	−0.008 (3)	−0.004 (3)	0.000 (3)
C22	0.051 (3)	0.069 (3)	0.049 (3)	0.009 (3)	0.004 (2)	−0.001 (2)
C18	0.060 (3)	0.089 (4)	0.066 (3)	−0.005 (3)	−0.002 (3)	0.003 (3)
C19	0.071 (4)	0.105 (5)	0.091 (5)	−0.009 (4)	0.007 (3)	0.034 (4)
C20	0.081 (4)	0.160 (8)	0.066 (4)	0.022 (5)	0.009 (3)	0.039 (5)
C21	0.075 (4)	0.165 (8)	0.059 (4)	0.009 (5)	−0.015 (3)	0.020 (5)
C16	0.219 (10)	0.093 (6)	0.082 (5)	0.028 (7)	0.018 (6)	0.008 (4)
N4	0.215 (9)	0.093 (5)	0.134 (6)	−0.017 (6)	−0.018 (6)	−0.008 (4)
N2	0.358 (15)	0.095 (6)	0.100 (6)	−0.006 (9)	−0.011 (8)	0.019 (5)

### Geometric parameters (Å, °)

**Table:**

O2—C8	1.437 (7)	C4—H4	0.9300
O2—C9	1.442 (8)	C23—C24	1.501 (8)
O3—C24	1.421 (7)	C7—C8	1.504 (8)
O3—C25	1.473 (8)	C9—C8	1.477 (7)
O4—C23	1.218 (7)	C9—H9	0.9800
N3—C23	1.356 (7)	C8—H8	0.9800
N3—C22	1.412 (5)	C25—C24	1.459 (8)
N3—H22	0.88 (2)	C25—C30	1.493 (7)
C10—C11	1.3900	C25—H25	0.9800
C10—C15	1.3900	C24—H24	0.9800
C10—C9	1.493 (7)	C32—N4	1.147 (10)
C11—C12	1.3900	C32—C28	1.356 (7)
C11—H11	0.9300	C31—C26	1.3900
C12—C13	1.3900	C31—C28	1.3900
C12—H12	0.9300	C31—H31	0.9300
C13—C16	1.362 (7)	C26—C30	1.3900
C13—C14	1.3900	C26—H26	0.9300
C14—C15	1.3900	C30—C27	1.3900
C14—H14	0.9300	C27—C29	1.3900
C15—H15	0.9300	C27—H27	0.9300
O1—C7	1.207 (6)	C29—C28	1.3900
N1—C7	1.328 (7)	C29—H29	0.9300
N1—C6	1.398 (5)	C17—C22	1.3900
N1—H6	0.90 (3)	C17—C21	1.3900
C1—C2	1.3900	C17—H17	0.9300
C1—C6	1.3900	C22—C18	1.3900
C1—H1	0.9300	C18—C19	1.3900
C2—C3	1.3900	C18—H18	0.9300
C2—H2	0.9300	C19—C20	1.3900
C3—C5	1.3900	C19—H19	0.9300
C3—H3	0.9300	C20—C21	1.3900
C5—C4	1.3900	C20—H20	0.9300
C5—H5	0.9300	C21—H21	0.9300
C4—C6	1.3900	C16—N2	1.147 (10)
			
C8—O2—C9	61.7 (4)	O2—C8—C9	59.3 (4)
C24—O3—C25	60.5 (4)	O2—C8—C7	119.4 (5)
C23—N3—C22	126.1 (4)	C9—C8—C7	121.0 (5)
C23—N3—H22	121 (3)	O2—C8—H8	115.2
C22—N3—H22	111 (3)	C9—C8—H8	115.2
C11—C10—C15	120.0	C7—C8—H8	115.2
C11—C10—C9	119.1 (3)	C24—C25—O3	58.0 (4)
C15—C10—C9	120.6 (3)	C24—C25—C30	125.3 (5)
C10—C11—C12	120.0	O3—C25—C30	117.2 (5)
C10—C11—H11	120.0	C24—C25—H25	114.7
C12—C11—H11	120.0	O3—C25—H25	114.7
C13—C12—C11	120.0	C30—C25—H25	114.7
C13—C12—H12	120.0	O3—C24—C25	61.5 (4)
C11—C12—H12	120.0	O3—C24—C23	116.6 (5)
C16—C13—C14	119.2 (6)	C25—C24—C23	119.9 (6)
C16—C13—C12	120.8 (6)	O3—C24—H24	115.9
C14—C13—C12	120.0	C25—C24—H24	115.9
C15—C14—C13	120.0	C23—C24—H24	115.9
C15—C14—H14	120.0	N4—C32—C28	178.0 (11)
C13—C14—H14	120.0	C26—C31—C28	120.0
C14—C15—C10	120.0	C26—C31—H31	120.0
C14—C15—H15	120.0	C28—C31—H31	120.0
C10—C15—H15	120.0	C31—C26—C30	120.0
C7—N1—C6	125.5 (4)	C31—C26—H26	120.0
C7—N1—H6	110 (3)	C30—C26—H26	120.0
C6—N1—H6	113 (3)	C26—C30—C27	120.0
C2—C1—C6	120.0	C26—C30—C25	123.4 (4)
C2—C1—H1	120.0	C27—C30—C25	116.5 (4)
C6—C1—H1	120.0	C29—C27—C30	120.0
C1—C2—C3	120.0	C29—C27—H27	120.0
C1—C2—H2	120.0	C30—C27—H27	120.0
C3—C2—H2	120.0	C27—C29—C28	120.0
C5—C3—C2	120.0	C27—C29—H29	120.0
C5—C3—H3	120.0	C28—C29—H29	120.0
C2—C3—H3	120.0	C32—C28—C29	119.2 (5)
C3—C5—C4	120.0	C32—C28—C31	120.6 (5)
C3—C5—H5	120.0	C29—C28—C31	120.0
C4—C5—H5	120.0	C22—C17—C21	120.0
C6—C4—C5	120.0	C22—C17—H17	120.0
C6—C4—H4	120.0	C21—C17—H17	120.0
C5—C4—H4	120.0	C18—C22—C17	120.0
C4—C6—C1	120.0	C18—C22—N3	119.9 (3)
C4—C6—N1	124.0 (3)	C17—C22—N3	120.1 (3)
C1—C6—N1	116.0 (3)	C19—C18—C22	120.0
O4—C23—N3	125.2 (6)	C19—C18—H18	120.0
O4—C23—C24	118.8 (5)	C22—C18—H18	120.0
N3—C23—C24	115.4 (5)	C20—C19—C18	120.0
O1—C7—N1	125.2 (5)	C20—C19—H19	120.0
O1—C7—C8	119.7 (5)	C18—C19—H19	120.0
N1—C7—C8	114.9 (5)	C19—C20—C21	120.0
O2—C9—C8	59.0 (4)	C19—C20—H20	120.0
O2—C9—C10	117.3 (5)	C21—C20—H20	120.0
C8—C9—C10	121.4 (5)	C20—C21—C17	120.0
O2—C9—H9	115.7	C20—C21—H21	120.0
C8—C9—H9	115.7	C17—C21—H21	120.0
C10—C9—H9	115.7	N2—C16—C13	178.2 (11)
			
C15—C10—C11—C12	0.0	C24—O3—C25—C30	116.4 (6)
C9—C10—C11—C12	173.8 (4)	C25—O3—C24—C23	−111.2 (6)
C10—C11—C12—C13	0.0	C30—C25—C24—O3	−102.6 (7)
C11—C12—C13—C16	179.8 (6)	O3—C25—C24—C23	106.0 (6)
C11—C12—C13—C14	0.0	C30—C25—C24—C23	3.5 (10)
C16—C13—C14—C15	−179.8 (6)	O4—C23—C24—O3	163.3 (6)
C12—C13—C14—C15	0.0	N3—C23—C24—O3	−24.7 (9)
C13—C14—C15—C10	0.0	O4—C23—C24—C25	92.4 (8)
C11—C10—C15—C14	0.0	N3—C23—C24—C25	−95.6 (7)
C9—C10—C15—C14	−173.7 (4)	C28—C31—C26—C30	0.0
C6—C1—C2—C3	0.0	C31—C26—C30—C27	0.0
C1—C2—C3—C5	0.0	C31—C26—C30—C25	−175.9 (4)
C2—C3—C5—C4	0.0	C24—C25—C30—C26	54.8 (8)
C3—C5—C4—C6	0.0	O3—C25—C30—C26	−13.7 (7)
C5—C4—C6—C1	0.0	C24—C25—C30—C27	−121.2 (6)
C5—C4—C6—N1	177.7 (4)	O3—C25—C30—C27	170.3 (4)
C2—C1—C6—C4	0.0	C26—C30—C27—C29	0.0
C2—C1—C6—N1	−177.8 (4)	C25—C30—C27—C29	176.2 (4)
C7—N1—C6—C4	−27.2 (7)	C30—C27—C29—C28	0.0
C7—N1—C6—C1	150.6 (5)	N4—C32—C28—C29	66 (29)
C22—N3—C23—O4	−2.3 (10)	N4—C32—C28—C31	−110 (29)
C22—N3—C23—C24	−173.7 (5)	C27—C29—C28—C32	−175.8 (5)
C6—N1—C7—O1	−10.1 (10)	C27—C29—C28—C31	0.0
C6—N1—C7—C8	175.3 (5)	C26—C31—C28—C32	175.8 (6)
C8—O2—C9—C10	111.9 (5)	C26—C31—C28—C29	0.0
C11—C10—C9—O2	179.7 (4)	C21—C17—C22—C18	0.0
C15—C10—C9—O2	−6.5 (6)	C21—C17—C22—N3	−178.8 (4)
C11—C10—C9—C8	−111.7 (5)	C23—N3—C22—C18	142.3 (5)
C15—C10—C9—C8	62.1 (7)	C23—N3—C22—C17	−38.9 (7)
C9—O2—C8—C7	−110.7 (6)	C17—C22—C18—C19	0.0
C10—C9—C8—O2	−105.1 (6)	N3—C22—C18—C19	178.8 (4)
O2—C9—C8—C7	108.0 (6)	C22—C18—C19—C20	0.0
C10—C9—C8—C7	2.9 (9)	C18—C19—C20—C21	0.0
O1—C7—C8—O2	166.4 (6)	C19—C20—C21—C17	0.0
N1—C7—C8—O2	−18.7 (8)	C22—C17—C21—C20	0.0
O1—C7—C8—C9	96.6 (8)	C14—C13—C16—N2	−82 (44)
N1—C7—C8—C9	−88.5 (7)	C12—C13—C16—N2	98 (44)

### Hydrogen-bond geometry (Å, °)

**Table:**

*D*—H···*A*	*D*—H	H···*A*	*D*···*A*	*D*—H···*A*
N1—H6···O1^i^	0.90 (4)	2.21 (3)	2.960 (6)	141 (4)
N3—H22···O4^ii^	0.89 (3)	2.09 (3)	2.923 (5)	157 (4)
C8—H8···O3^iii^	0.98	2.49	3.287 (8)	138
C15—H15···O1^i^	0.93	2.58	3.505 (6)	171
C24—H24···O2^iv^	0.98	2.54	3.370 (7)	142

Symmetry codes: (i) *x*−1, *y*, *z*; (ii) *x*+1, *y*, *z*; (iii) −*x*+2, *y*−1/2, −*z*+2; (iv) −*x*+1, *y*+1/2, −*z*+2.
